# Supplementary material for: Raspberry Viruses in the Czech Republic, with Identification of a Novel Virus: Raspberry Virus A
Source: Viruses. 2025 Dec 9;17(12):1597. doi: 10.3390/v17121597 (PMC12737478; doi:10.3390/v17121597)
Supplement: Supplementary file 1 [file viruses-17-01597-s001.zip › Supplementary Figures.pdf]

Supplementary Figures

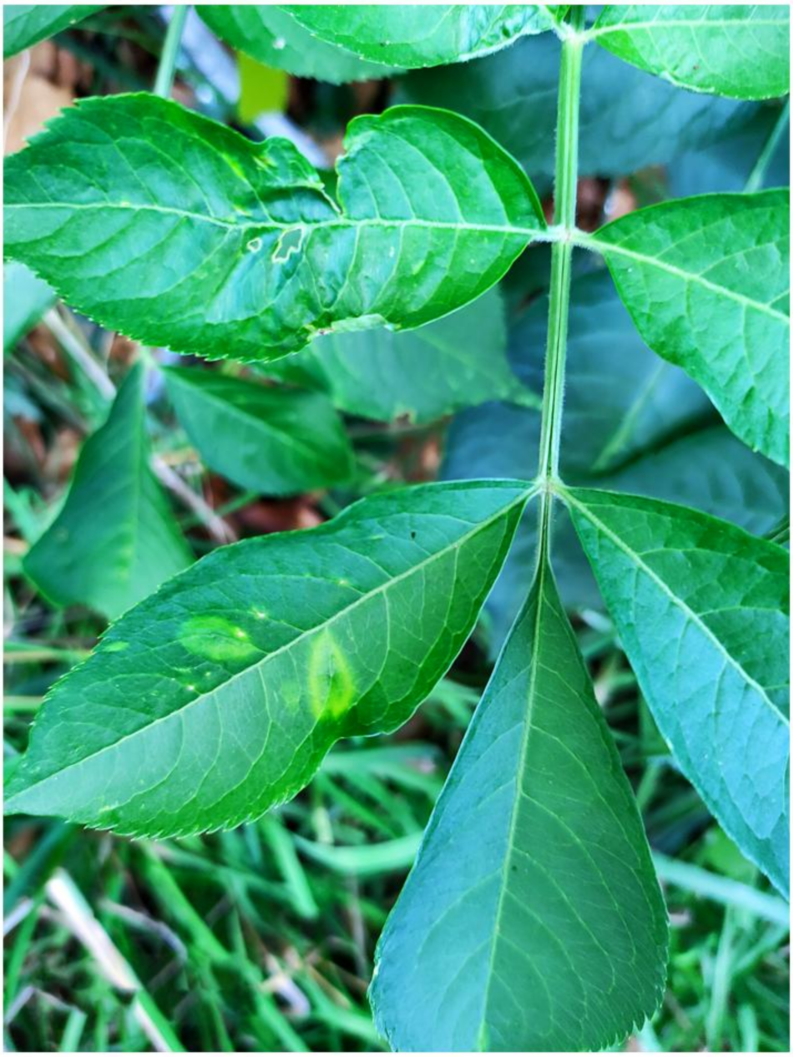

Figure S1. Leaf of *Sambucus nigra* showing prominent yellow rings and chlorotic dots infected with raspberry bushy dwarf virus, elderberry virus A, and elderberry virus B.

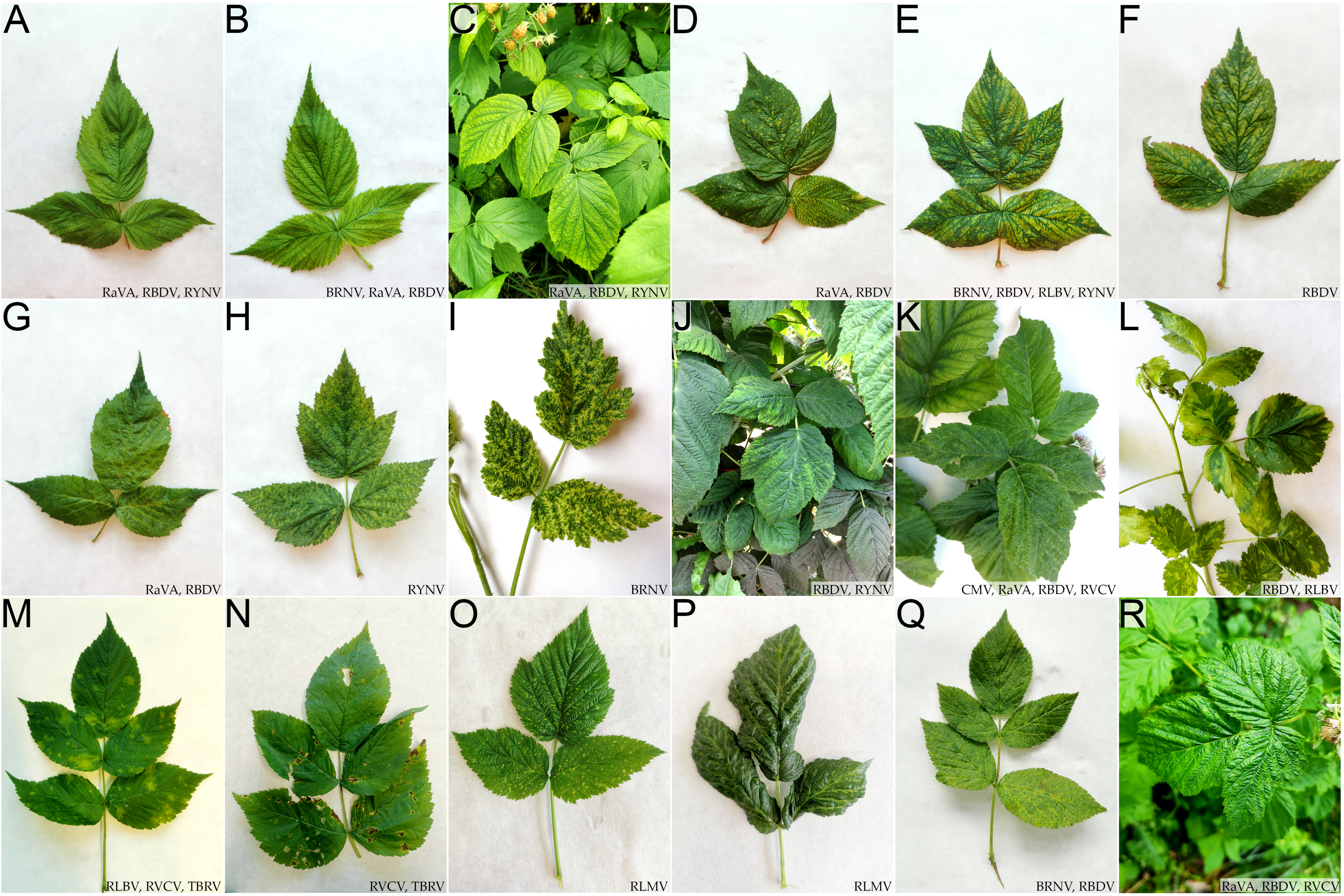

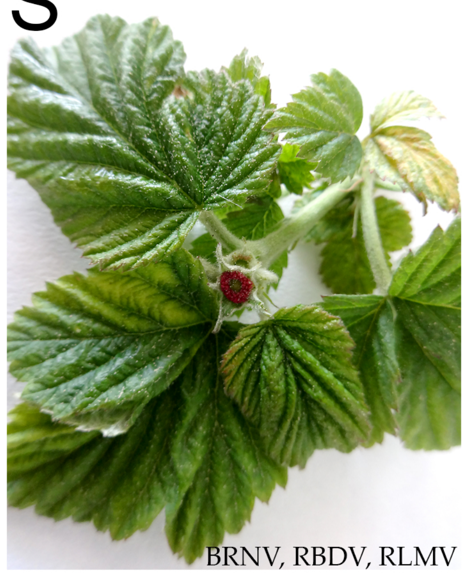

Figure S2. Symptoms observed on leaves and shoots of raspberry plants infected with different viruses. Each panel is labeled with the viruses detected in the corresponding sample. (A) B45, cv. Polka, light green areas around veins; (B) B51, cv. Polka, slight yellowing; (C) B48, cv. Polka, yellowing; (D) B43, cv. Polka, yellowing; (E) B39, cv. Polka, severe yellowing, dark green veins; (F) B54, cv. Polka, severe yellowing/reddening, dark green veins, small necrosis; (G) B52, cv. Polka, veinal chlorotic mottle with necrosis; (H) B65, cv. Sugana, mosaic; (I) A702, wild raspberry, severe mosaic; (J) A998, cv. Endosadira, dark green mosaic; (K) A806, cv. Canby, vein clearing, yellowing; (L) A928, cv. unknown, severe leaf blotch; (M) A982, wild raspberry, severe diffusion lesions; (N) A983, wild raspberry, severe necrotic lesions with yellow halo, perforations; (O) B14, cv. Enrosadira, dot mosaic; (P) B21, cv. Enrosadira, pattern around veins, curling, necrosis; (Q) B34, wild raspberry, veinal chlorotic mottle; (R) B363, cv. Canby, curling, mosaic, slight vein clearing; and (S) B313, cv. Canby, flower malformation, yellowing/reddening, dwarfism.
